# Vedolizumab induced acute interstitial nephritis

**DOI:** 10.1093/omcr/omaf148

**Published:** 2026-01-28

**Authors:** Darren Fernandes, Alok Mathew, Alastair Ferraro, Sunil Samuel

**Affiliations:** The Department of Gastroenterology, Nottingham Digestive Diseases Centre, Derby Road, Nottingham, NG7 2UH, United Kingdom; The Department of Gastroenterology, Nottingham Digestive Diseases Centre, Derby Road, Nottingham, NG7 2UH, United Kingdom; Renal and Transplant Unit, Nottingham University Hospitals, Hucknall Road, Nottingham, NG5 1PB, United Kingdom; The Department of Gastroenterology, Nottingham Digestive Diseases Centre, Derby Road, Nottingham, NG7 2UH, United Kingdom

**Keywords:** gastroenterology, nephrology

## Abstract

Vedolizumab is a gut selective anti-integrin monoclonal antibody with a very good and proven safety profile in the treatment of ulcerative colitis (UC). This case report highlights a rather unusual complication that every gastroenterologist ought to be aware of. It relates to the association of acute interstitial nephritis with vedolizumab usage in the management of UC. A 53-year-old man with liver cirrhosis due to Primary Sclerosing Cholangitis and UC was treated with vedolizumab following failed therapy with thiopurines and mesalazine. This led to improvement in his bowel function but his renal function steadily declined over subsequent months, in the presence of reactive urinary sediments. A subsequent renal biopsy demonstrated acute interstitial nephritis. Cessation of vedolizumab, along with high dose steroids treatment, led to near complete resolution of his renal failure.

## Introduction

The number of treatment options in inflammatory bowel disease (IBD), comprising Crohn’s disease (CD) and ulcerative colitis (UC), is continuously increasing. Anti-tumour necrosis factor (anti-TNF) agents have become the mainstay of therapy in patients failing conventional treatment. A significant number of patients with IBD either do not respond, lose response or do not tolerate anti-TNF treatment [1–4]. For these patients, as well as anti-TNF naïve ones, other treatment options include newer biological agents such as Vedolizumab. Vedolizumab is a humanised monoclonal antibody which binds the α4β7 integrin expressed on gut lymphocytes, and which inhibits their migration from the blood into the intestinal mucosa [5]. Compared with anti-TNF treatment, Vedolizumab is believed to have an overall lower risk of adverse drug reactions [6]. However, as a comparably new contribution to the advanced treatment options in IBD, less frequent and unexpected adverse drug reactions may unfold over time.

## Case report

**Figure 1:**
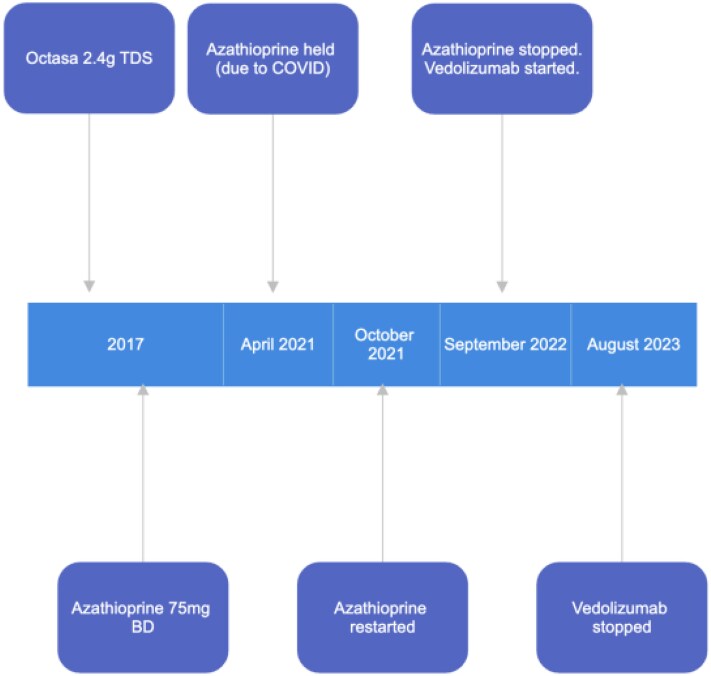
Showing timeline of treatments given for ulcerative colitis.

We present the case of a 53-year-old gentleman with liver cirrhosis due to primary sclerosing cholangitis (PSC) and ulcerative colitis (UC). He was previously treated with Mesalazine but it did not provide adequate symptom control. He had also been on Azathioprine, which was discontinued initially because of the COVID-19 pandemic but then later on due to low neutrophil and lymphocyte counts. In September 2022, he was started on Vedolizumab (Fig. 1), which appeared to improve his UC symptoms.

**Figure 2:**
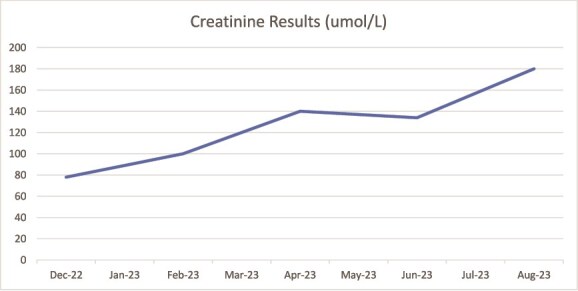
Showing rise in creatinine following commencement of Vedolizumab.

At that time, his baseline creatinine level was 78 μmol/l; however, it gradually rose to 180 μmol/l by August 2023 without any apparent precipitating factors (Fig. 2). Urine analysis revealed the presence of both blood and protein. An ultrasound of the kidneys and bladder showed no abnormalities, aside from a simple cyst in the left kidney.

**Figure 3:**
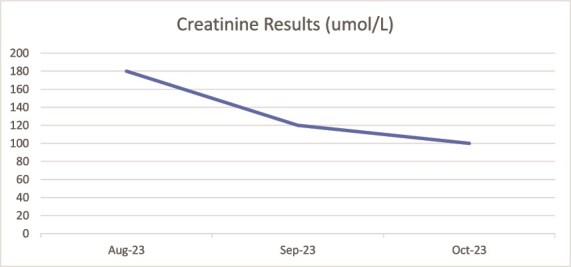
Showing creatinine results following corticosteroid therapy.

A renal biopsy revealed significant inflammatory infiltrates within the interstitium, along with notable interstitial fibrosis and tubular atrophy, and lesser findings attributable to his liver disease; this led to the diagnosis of interstitial nephritis likely related to Vedolizumab. Consequently, his Vedolizumab was discontinued and he was started on high-dose steroids. Over the subsequent six months his renal function improved and the steroids were weaned off (Fig. 3). The renal function has remained stable since then, but unfortunately, his UC began to flare and so he was started on Ustekinumab.

## Discussion

This report presents a very rare case of AIN associated with Vedolizumab therapy [7]. AIN is recognised as a significant contributor to acute kidney injury, accounting for up to 20% of all such cases [8]. Among the various causes of AIN, around 70% are linked to medications, while the remainder arise from autoimmune disorders, infections or are idiopathic in origin. The drugs most frequently implicated in AIN include antibiotics, non-steroidal anti-inflammatory drugs (NSAIDs) and proton pump inhibitors (PPIs). In patients with IBD, however, aminosalicylates are the most commonly identified culprits [9].

**Figure 4:**
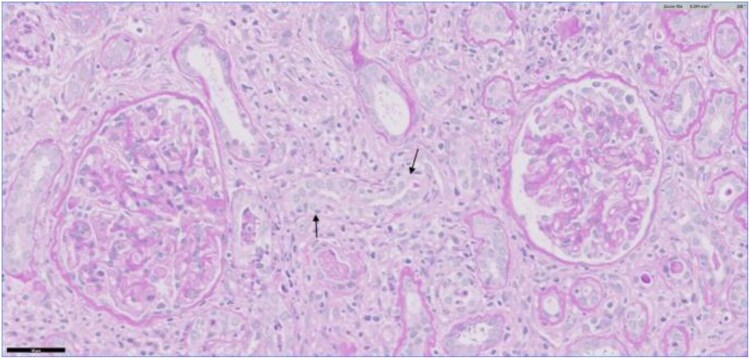
Showing infiltrate of lymphocytes and macrophages in the interstitium and lymphocytic tubulitis (arrows). (copied from Simpson et. al 2021 [10]).

AIN is marked by features such as interstitial inflammation, oedema and tubulitis, with a notable presence of CD4+ T lymphocytes and other mononuclear cells, along with varying quantities of eosinophils (Fig. 4) [10]. Early identification of AIN is essential, as it has the potential to progress to chronic kidney disease (CKD), driven by fibroblast activation that results in interstitial fibrosis and tubular atrophy [5].

The classic clinical presentation of drug-induced interstitial nephritis often involves a triad of symptoms: fever, eosinophilia and rash, which typically arise within a few days of starting the problematic medication. However, this characteristic triad is observed in less than 10% of patients [8]. As demonstrated in the current case, the onset of drug-induced interstitial nephritis can be significantly delayed, occurring weeks or even months after the initiation of the offending agent.

The cornerstone of managing AIN is the discontinuation of the drug responsible for the condition [10]. If there is no notable improvement within three to five days following cessation, high-dose corticosteroids are generally initiated. Early intervention with corticosteroids is thought to be particularly effective because the early interstitial infiltrates are typically responsive to steroid treatment [10]. Prompt recognition and treatment of drug-induced interstitial nephritis correlate with a lower risk of developing chronic kidney disease [10]. A definitive diagnosis often necessitates a renal biopsy, which should be carried out promptly to guide management. The extent of interstitial fibrosis observed during biopsy is closely linked to an increased risk of chronic renal impairment; patients with evidence of established interstitial fibrosis are less likely to respond positively to steroid therapy [9]. Studies have shown that patients who receive steroid therapy within two weeks of stopping the offending medication are more likely to recover their baseline renal function than those who experience delays, averaging around 34 days before steroid initiation [11].

In the case under discussion, our patient was not taking any additional medications that could have contributed to interstitial nephritis. At the time of his presentation, he was in both clinical and biochemical remission from his IBD, leading us to consider extraintestinal manifestations of his disease as being unlikely. Furthermore, whilst ANCA-associated vasculitis (AAV) does rarely cause interstitial nephritis, there are numerous features which make that an unlikely diagnosis here: the gentleman had a history of PR3 positivity, but no prior ANCA-related clinical disease; there were no glomerular features of AAV despite this being its usual renal presentation; and his renal disease has not relapsed despite withdrawal of steroids (and no use of other ANCA-treating agents). Thus, whilst the patient was not rechallenged with Vedolizumab to definitively confirm it caused the AIN [12], our comprehensive evaluation suggests that AIN secondary to Vedolizumab was the most likely explanation for his condition. This case highlights the critical need for heightened awareness regarding drug-induced AIN, particularly in patients undergoing treatment with biological agents for inflammatory bowel disease.
